# Supplementary material for: HCLC-FC: A novel statistical method for phenome-wide association studies
Source: PLoS One. 2022 Nov 9;17(11):e0276646. doi: 10.1371/journal.pone.0276646 (PMC9645610; doi:10.1371/journal.pone.0276646)
Supplement: S1 File — (DOCX) [file pone.0276646.s001.docx]

**Supplementary Materials**

HCLC-FC: a novel statistical method for phenome-wide association studies

Xiaoyu Liang^2^, Xuewei Cao^1^, Qiuying Sha^1^, Shuanglin Zhang^1*^

^1^ Department of Mathematical Sciences, Michigan Technological University, Houghton, Michigan, United States of America

^2^ Department of Preventive Medicine, Division of Biostatistics, University of Tennessee Health Science Center, Memphis, Tennessee, United States of America

*** Corresponding author**

E-mail: [shuzhang@mtu.edu](mailto:shuzhang@mtu.edu) (SZ)


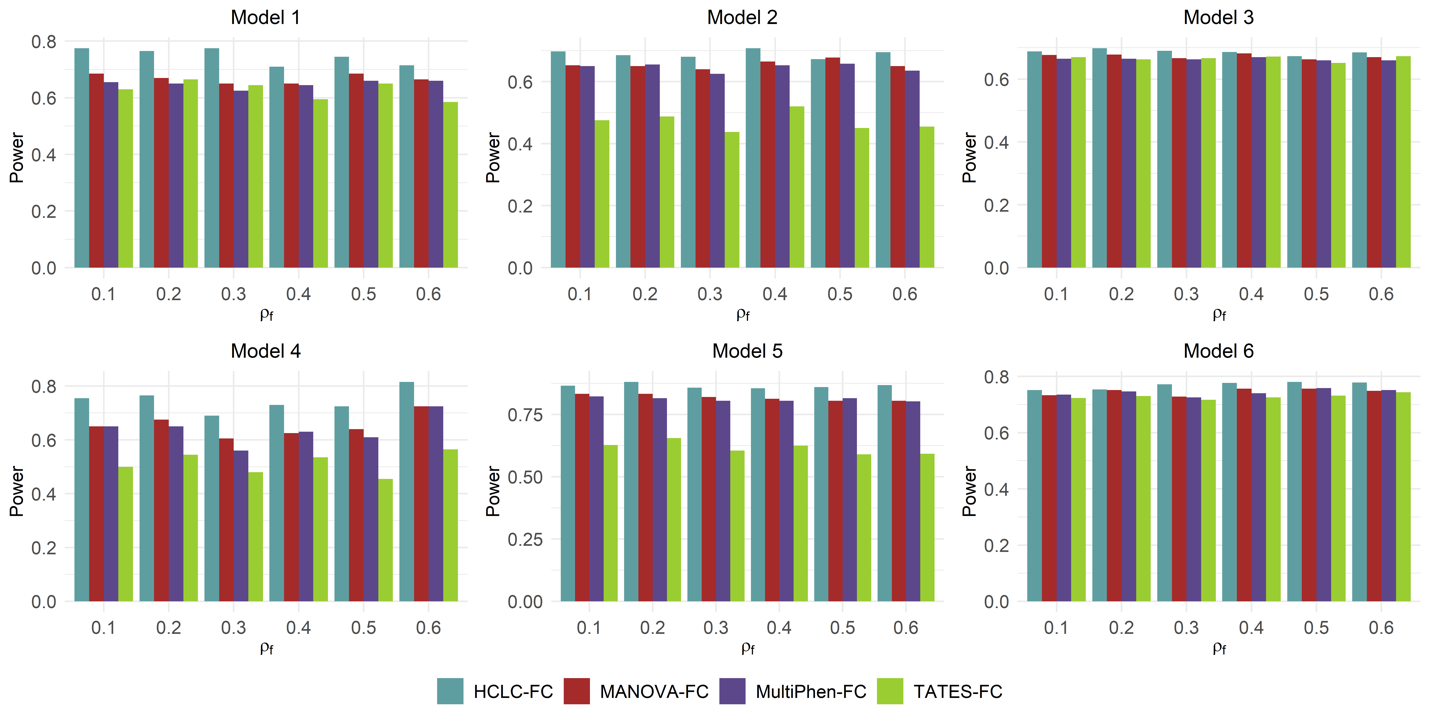


**Fig S1. Power comparisons of the four tests for the power as a function of correlation between phenotypic categories (**$\boldsymbol{\rho}_{\boldsymbol{f}}$**) under the six models for 1,000 phenotypes (**$\boldsymbol{K=1,000}$**).** MAF is 0.3. The sample size ($n$) is 2,000. $\rho_{e}=0.3$ and $c^{2}=0.5$. The effect sizes of the six models are 0.014, 0.060, 0.090, 0.006, 0.060, and 0.090. The power of the four tests is evaluated using 200 replicated samples at a nominal FDR level of 5%.


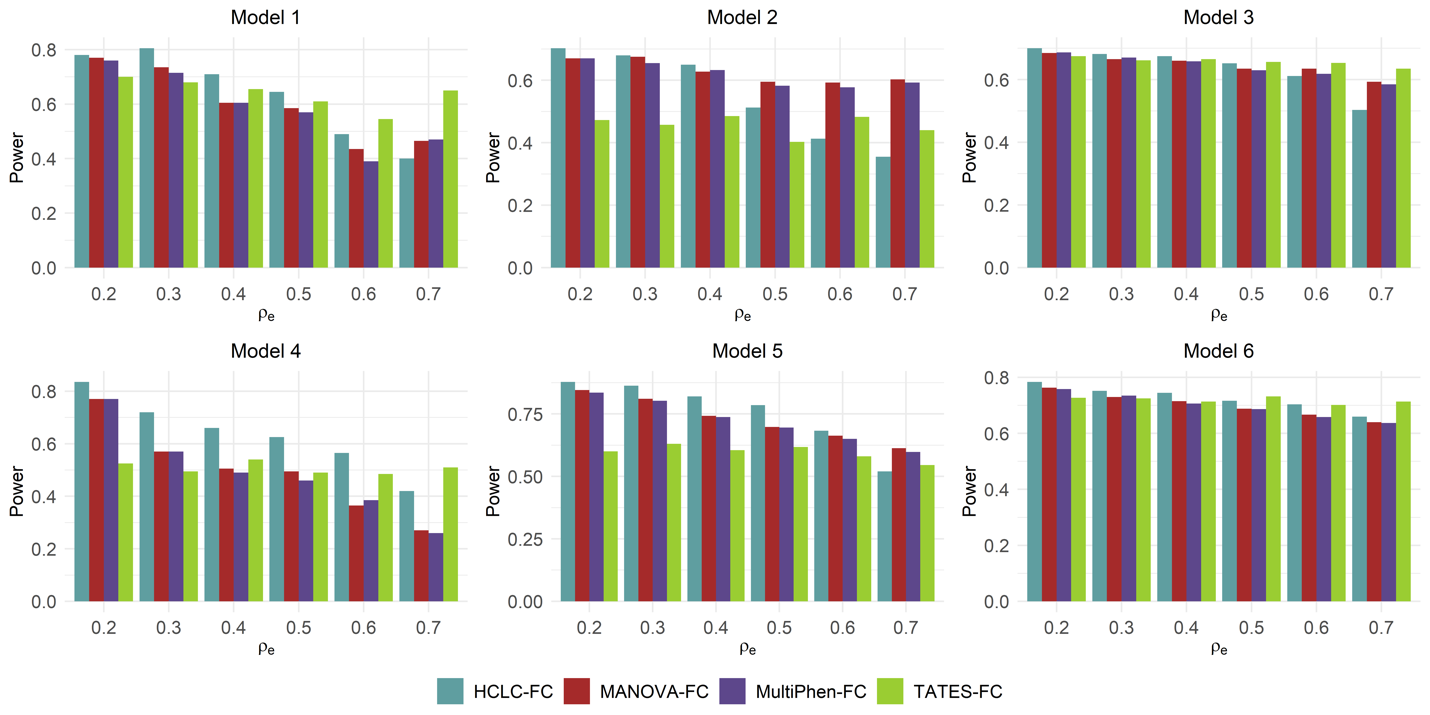


**Fig S2. Power comparisons of the four tests for the power as a function of correlation within each phenotypic category (**$\boldsymbol{\rho}_{\boldsymbol{e}}$**) under the six models for 1,000 phenotypes (**$\boldsymbol{K=1,000}$**).** MAF is 0.3. The sample size ($n$) is 2,000. $\rho_{f}=0.2$ and $c^{2}=0.5$. The effect sizes of the six models are 0.014, 0.060, 0.090, 0.006, 0.060, and 0.090. The power of the four tests is evaluated using 200 replicated samples at a nominal FDR level of 5%.


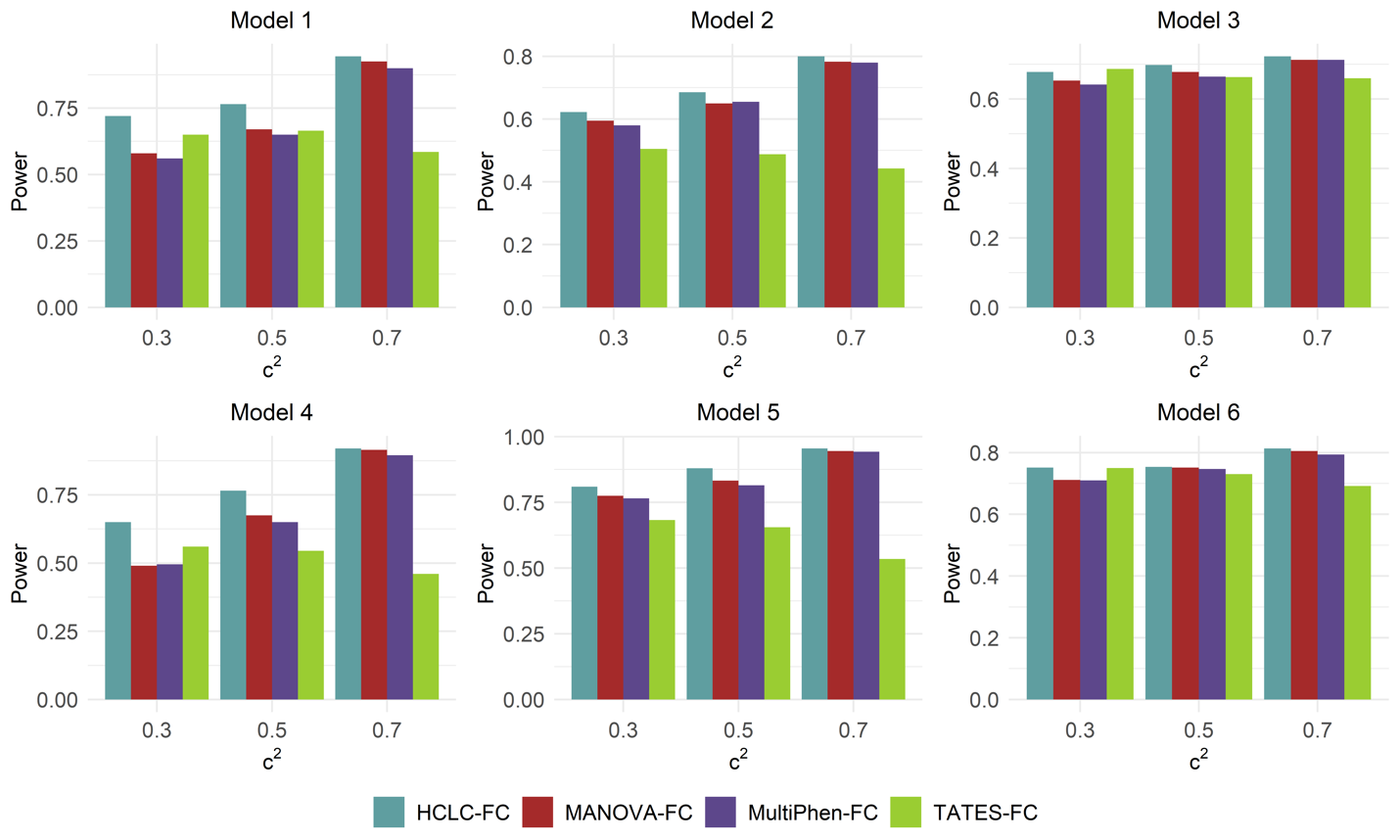


**Fig S3. Power comparisons of the four tests for the power as a function of the constant** $\boldsymbol{c}^{\boldsymbol{2}}$ **under the six models for 1,000 phenotypes (**$\boldsymbol{K=1,000}$**).** MAF is 0.3. The sample size ($n$) is 2,000. $\rho_{f}=0.2$ and $\rho_{e}=0.3$. The effect sizes of the six models are 0.014, 0.060, 0.090, 0.006, 0.060, and 0.090. The power of the four tests is evaluated using 200 replicated samples at a nominal FDR level of 5%.


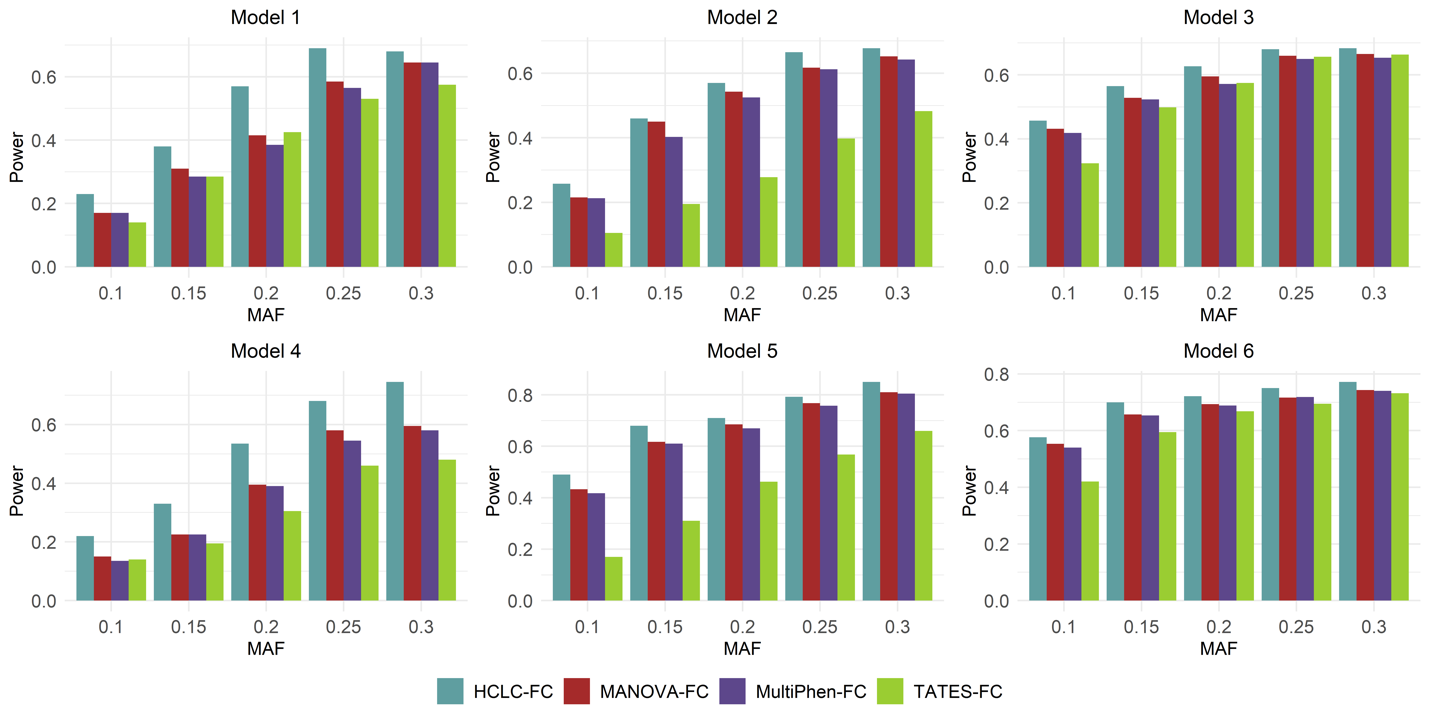


**Fig S4 Power comparisons of the four tests for the power as a function of minor allele frequency (MAF) under the six models for 1,000 phenotypes (**$\boldsymbol{K=1,000}$**).** The sample size ($n$) is 2,000. $\rho_{f}=0.2$, $\rho_{e}=0.3$ and $c^{2}=0.5$. The effect sizes of the six models are 0.014, 0.060, 0.090, 0.006, 0.060, and 0.090. The power of the four tests is evaluated using 200 replicated samples at a nominal FDR level of 5%.


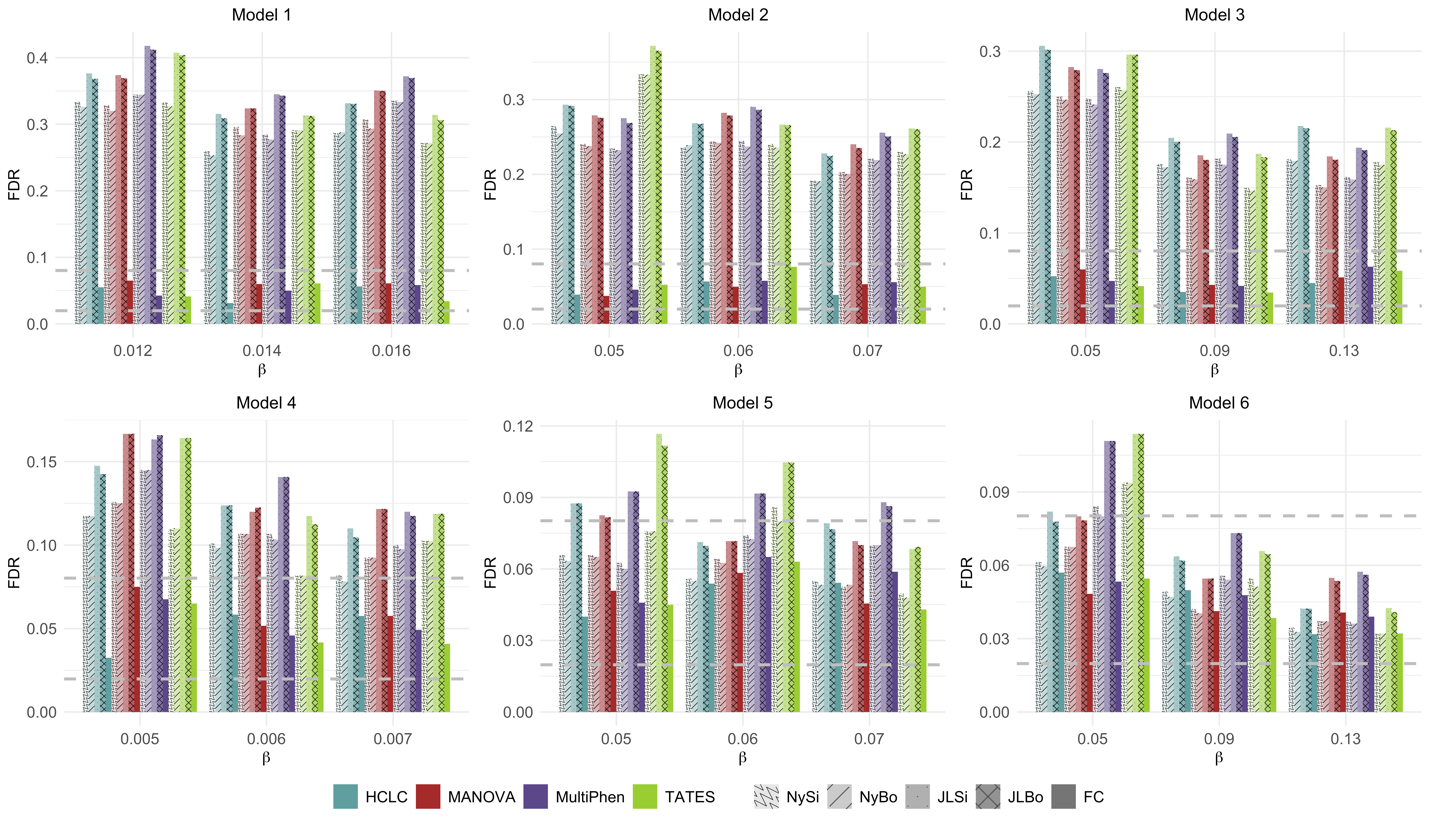


**Fig S5. FDR comparisons of the four tests combined with five FDR control methods for the FDR as a function of effect size (**$\boldsymbol{\beta}$**) under the six models for 1,000 phenotypes (**$\boldsymbol{K=1,000}$**).** MAF is 0.3. The sample size ($n$) is 2,000. $\rho_{f}=0.2$, $\rho_{e}=0.3$, and $c^{2}=0.5$. The power of all of the four tests is evaluated using 200 replicated samples at a nominal FDR level of 5%. The grey dash lines represent the 95% confidence interval [0.0198 0.0802]. NySi: Nyholt spectral decomposition method combining with Sidak multiple test correction; NyBo: Nyholt spectral decomposition method combining with Bonferroni correction; JiSi: Li and Ji method combining with Sidak multiple test correction; JiBo: Li and Ji method combining with Bonferroni correction.


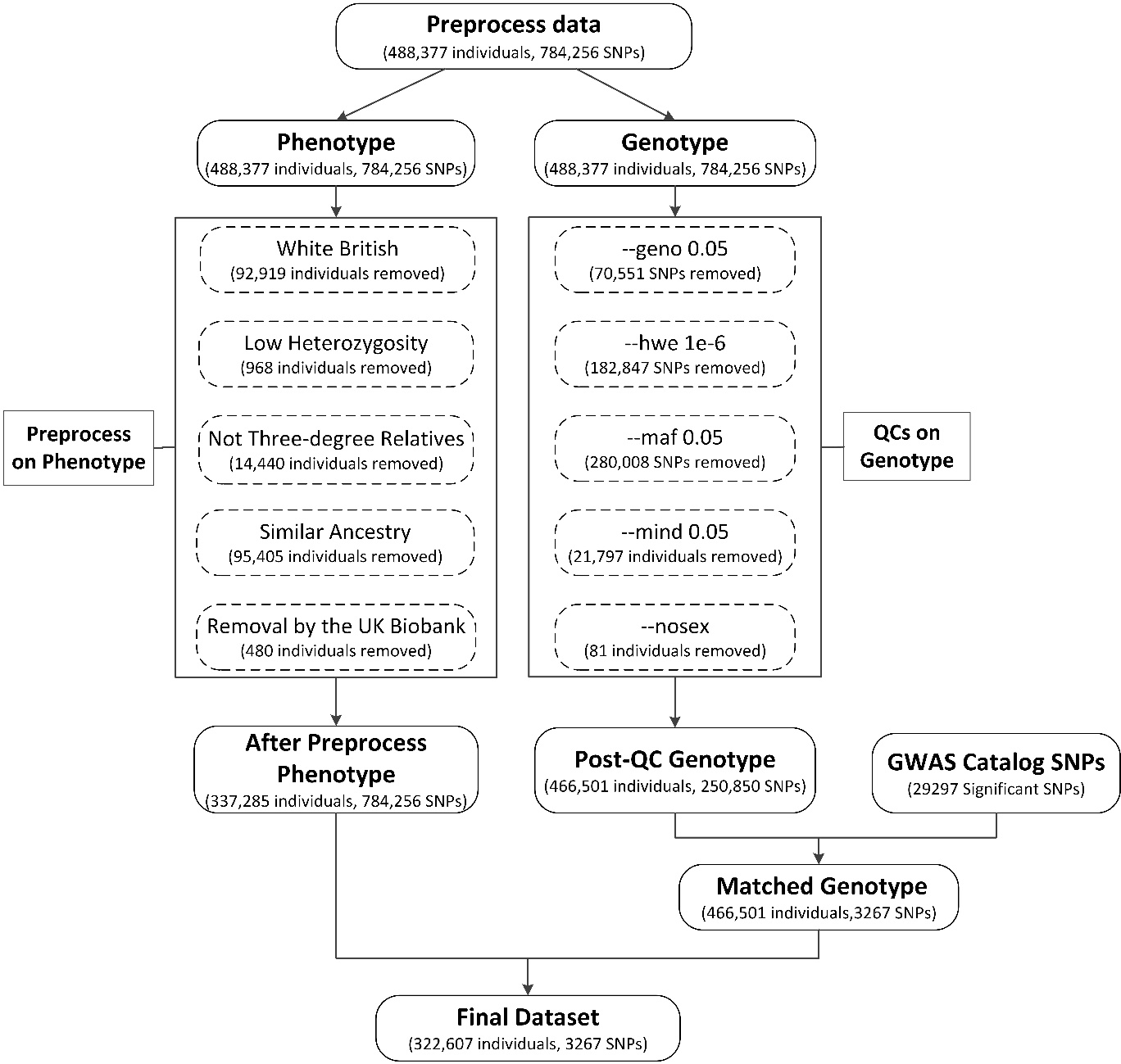


**Fig S6. Flow chart of UK Biobank data preprocessing.** *Preprocess on phenotype*: i. Select White British subjects (White British); ii. Remove individuals who are marked as outliers for heterozygosity or missing rates (Low Heterozygosity); iii. Exclude individuals who have been identified to have ten or more third-degree relatives or closer (Not Three-degree Relatives); iv. Remove individuals having very similar ancestry based on the principal component analysis of the genotypes (Similar Ancestry); v. Remove individuals that are recommended for removal by the UK Biobank (Removal by the UK Biobank). *Quality controls (QCs) on genotype*: Filter out genetic variants, with i. Missing rate larger than 5% (“--mind 0.05”), ii. Hardy-Weinberg equilibrium exact test p-values less than ${10}^{-6}$ (“--hwe 1e-6”), iii. Minor allele frequency (MAF) less than 5% (“--maf 0.05”). We also filter out individuals, with iv. Missing rate larger than 5% (“--mind 0.05”) v. Individuals without sex (“--no-sex”).


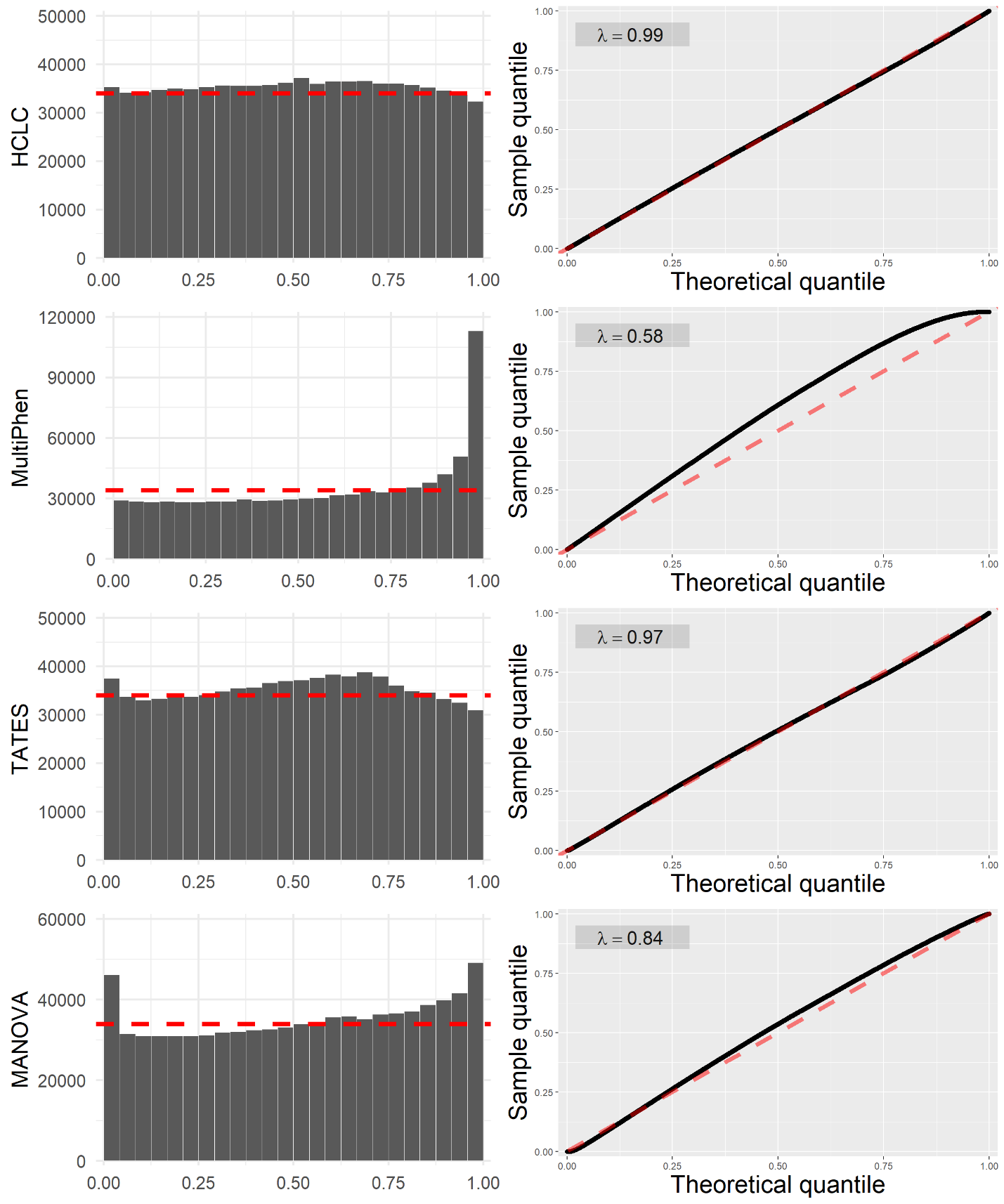


**Fig S7. The histogram of p-values (left) and QQ plot for uniform distribution of each method (right) based on 849,420 replicated samples.** The red dashed line represents the theoretical frequency and quantile for the standard uniform distribution.


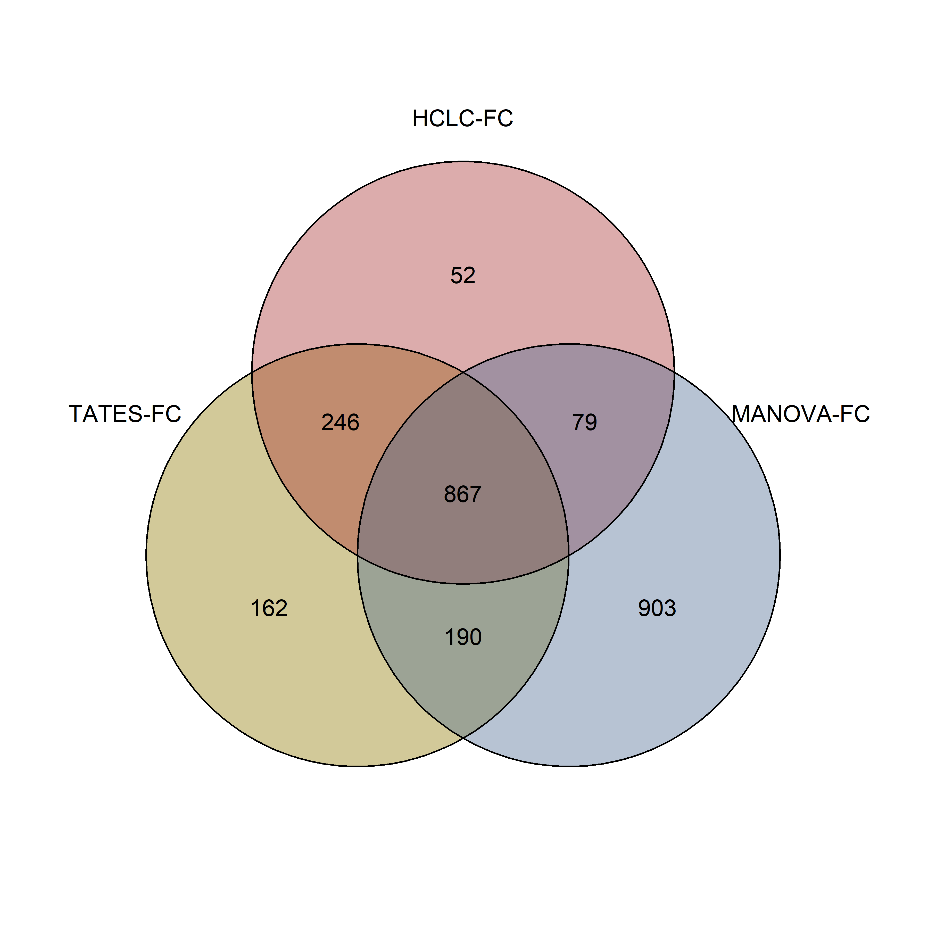


**Fig S8. Comparison of the identified SNPs using HCLC-FC, MANOVA-FC, and TATES-FC.**


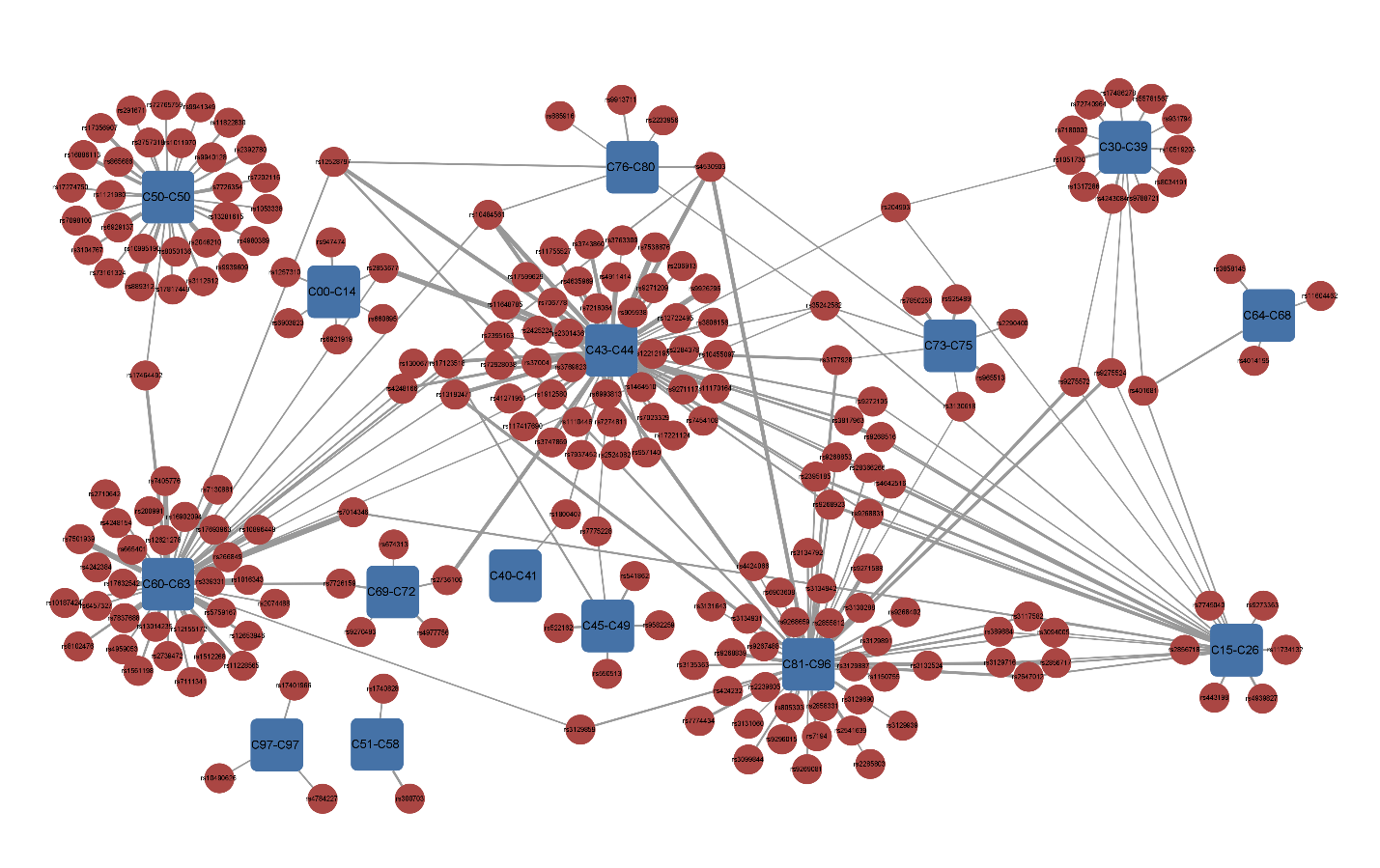


**Fig S9. The associations between SNPs and the malignant neoplasms phenotypic blocks identified by the HCLC-FC method.** The red circles represent SNPs, and the blue squares represent 15 malignant neoplasms phenotypic blocks C00-C97 (C00-C14: Malignant neoplasms of lip, oral cavity and pharynx; C15-26: Malignant neoplasms of digestive organs; C30-C39: Malignant neoplasms of respiratory and intrathoracic organs; C40-C41: Malignant neoplasms of bone and articular cartilage; C43-C44: Melanoma and other malignant neoplasms of skin; C45-C49: Malignant neoplasms of mesothelial and soft tissue; C50-C50: Malignant neoplasm of breast; C51-C58: Malignant neoplasms of female genital organs; C60-C63: Malignant neoplasms of male genital organs; C64-C68: Malignant neoplasms of urinary tract; C69-C72: Malignant neoplasms of eye, brain and other parts of central nervous system; C73-C75: Malignant neoplasms of thyroid and other endocrine glands; C76-C80: Malignant neoplasms of ill-defined, secondary and unspecified sites; C81-C96: Malignant neoplasms, stated or presumed to be primary, of lymphoid, haematopoietic and related tissue; C97-C97: Malignant neoplasms of independent (primary) multiple sites). The width of the connection line represents the strength of association (-log10 scale p-value).
